# Mr. Traill, on Cold Affusion in the Late Influenza

**Published:** 1805-09-01

**Authors:** T. S Traill

**Affiliations:** Liverpool


### *To the Editors of the Medical and Physical Journal.*

GENTLEMEN,

IF the few following Observations on the Use of the cold Affusion in the Cure of the late Influenza, as it appeared in the Orkney Islands, are deemed worthy of a place in your excellent Journal, they are much at your service.

The exact period when this epidemic made its first appearance in those islands I am not able to state; but it certainly was not very prevalent there, till a considerable time after it had appeared in Paris, London, and even Edinburgh. In the course of the following summer I visited Edinburgh, and during a conversation with my friend Dr. Wright, I learned that he had treated influenza as a fever of debility, and had successfully employed the cold affusion; I mentioned my having employed the same remedy in this disease; and concluding that many other physicians had done the same, I abandoned the idea of transmitting an account of my practice to some of the Medical Journals. My attention to the subject was however roused by a careful perusal of the last edition of Dr. Currie's invaluable Reports on the Effects of cold Water in Febrile Diseases, where he mentions having employed the *tepid* affusion on himself while labouring under influenza, but says he did not employ the *cold* affusion, on account of the pulmonary symptoms and cough attending this disease. From this I conceive that the cold affusion, as a remedy in influenza, has been generally deemed a dangerous practice, and therefore a narrative of its successful employment may not be uninteresting.

The first case I was called to, was a woman aged 68 or 69, who complained of the usual languor, uneasiness, &c. that accompany febrile diseases, to which were superadded cough, difficulty of breathing, and considerable pain in the chest. The heat of the body was not very great; the pulse frequent, but not full; her belly costive. Before I was called, she was, at her own desire, bled to eight eight ounces, but without any abatement of the symptoms. Never having seen the disease, I perhaps might have prescribed bleeding, had not this been recently done without success, and had I not been previously warned by my correspondents, of the fatal consequences that too often followed this practice in influenza.

At Paris, as a friend informed me, the great mortality arose chiefly from venesection, to which the French are in many diseases too much addicted; and both at London, and at Edinburgh, it was said, bleeding had been attended with the worst consequences. Conceiving this to be a case of influenza, I determined not to have recourse to the lancet, but ordered a blister to be applied to the sternum, an enema to be administered, and small doses of Dover's powders to be given, both to promote perspiration, and by the opium it contained, to support the vis vitæ, and to relieve the cough. *Second morning.*—Blister rose well; pain of breast rather less urgent; pulse very frequent, and more feeble. Slight perspiration during the night. Had some sleep; enema operated once, and she had one natural stool this morning. Had I been aware that the moderate use of wine had been recommended by Dr. Wright, I should certainly have employed it; but I was intimidated from its use by the pulmonary complaints. Saw her again at night; breathing and pain of chest as in the morning; debility extreme. She expired easily towards morning.

The age of this patient no doubt was much against her recovery; but taking the rapid sinking of her strength, and other circumstances into consideration, I was of opinion, that the bleeding at least hastened her death; and I resolved not to employ the lancet in similar cases, should they occur.

A short time after this, the influenza became common in the town of Kirkwall, and spread with astonishing rapidity through the islands; but in rather a milder form than it assumed in France, London, or Edinburgh. It proved fatal however to several individuals, especially those advanced in life. A great many cases fell under my observation; and by prescribing, in the beginning of the disease, emetics of tartarized antimony, and afterwards by the use of gentle diaphoretics, calomel purges, and when the cough and pain of chest were urgent, blisters to the breast, I was so fortunate as to see all my patients recover; some of them indeed but very slowly, from the extraordinary debility which this singular disease induces.

The following accident first suggested to me a more

 decisive decisive mode of treatment, which I invariably found safe and advantageous.

From the smallness of many of the Orkney Islands, medical assistance can only be had by applying to the practitioners in Kirkwall or Stromness.

In an island four miles from Kirkwall, a boy of about thirteen years of age, was seized with symptoms of influenza; and to save the expence of calling out a medical man, his friends carried him by boat to town. When I was called to him, I found all the usual symptoms that characterize the disease, pretty strongly marked; and from his having been for a considerable time exposed to the weather in a cold, raw day, as well as from his having been during the passage accidentally wet with spray of the sea, I was apprehensive that the disease might terminate fatally; I therefore judged it expedient, both on my own account and theirs, to inform his parents, that my prognosis was unfavourable. But to my infinite surprize, he recovered more rapidly than any other patient in whom the symptoms were so violent. This led me immediately to bathe the arms, face, and hands of my other patients labouring under influenza, with cold water. This practice was to them highly grateful, and observing no bad consequences following, I thought myself justified, from what I had seen, to employ a more decisive application of cold to the surface of the body.

The first case I employed the cold affusion in was the following:

G. Mowat, aged sixteen, while attending some chemical processes, in which I was at that time engaged, complained of pain in his back, listlessness, vertigo, some pain in his breast, attended with fits of coughing, and such weakness that he was scarcely able to stand. His pulse was 110, and pretty strong; skin dry; tongue slightly furred; belly natural. Not having noted the degree of heat the thermometer indicated, I am unable to state it accurately; but it was somewhat above natural. These symptoms had not been so severe as to attract his attention, till on getting out of bed in the morning; but they were much increased since that time. (It was now one o'clock afternoon.) I ordered him to go home immediately, and prescribed gr. ijss. tart. antimonii, which produced copious vomiting. After the sweat caused by the emetic had entirely subsided, I directed two pails of cold water to be at the same instant dashed over his naked body. In two or three hours after the affusion I saw him again. The pain of chest was not increased, his pulse was reduced to 102; copious perspiration had come on, and he thought himself refreshed. Hab. haust. anodyn. cum gutt. xx. h. s.

Second Day. *In the morning*, his pulse 86; slept well; skin almost natural; tongue still a little white; repetat. affus. aqua frigid. As he had not had a stool, I prescribed gr. iv. of calomel. *In the evening*, the febrile symptoms almost gone; powder operated once; repetat. affus. et haust. h. s.

Next day he was able to go about; and the following one, insisted on assisting me in my experiments as usual.

Two or three days after his recovery, his younger brother, and a sister several years older, were both attacked with influenza; but their symptoms were more violent than his. A similar treatment was adopted in their cases; and so rapid was their recovery, that though it was not till early on Friday morning I was first sent for, when I came the following Sunday to enquire for my patients, I was surprized to learn that they had thought themselves so far recovered as to go to church; nor did this rashness induce a relapse.

The mother of the above patients, a short time after their illness, had the same complaint; but from peculiar circumstances that attended influenza in her case, I did not use the cold affusion. Her recovery was more slow than theirs had been. She had diaphoretic. mistur. which I never had occasion to prescribe where the affusion was employed; for perspiration soon followed its use, and the patient was relieved.

This plan of treatment I pursued in every other case of the disease that came under my care. The notes I took on two cases, one of a woman aged twenty-six, the other of a man aged forty-seven, which from the great violence of the pulmonary complaints that attended, were interesting, and in which I successfully employed the cold affusion, I have not at present by me. But in the extensive practice I then had in this complaint, I am enabled to state, that I never observed any bad effects arise from the employment of the cold affusion; and when used in the beginning of the attack, it was uniformly highly beneficial.

Some practitioners may contend, that this practice might answer in the comparatively mild form the influenza assumed in Orkney, but would have been improper in the virulent cases that occurred in many great towns; but from the experience of its safety by Dr. Wright, and the application of the tepid affusion by Dr. Currie, in his

 own own case (where his consitution was unfavourable to any practice that might tend to increase the inflammation of the lungs) and from the above cases, we may at least be tempted to try the effects of the cold affusion in influenza, if ever it should visit us again; a circumstance not unlikely, since about twenty-two years ago, it was, I believe, fully as prevalent as lately over Europe.

Your's, with respect,

*Liverpool, July* 10, 1805. T. S. TRAILL.

